# Editorial ‐ A Professional Doctorate Degree in Medical Physics

**DOI:** 10.1120/jacmp.v9i2.2876

**Published:** 2008-05-08

The training of medical physicists is one of the “hot button” topics currently making the rounds in the North American medical physics community. Specifically, the topic of concern is the establishment of a professional doctorate (PD) degree in medical physics. The existence of such a graduate degree and its impact on the training of medical physicists in North America might have implications in the training of medical physicists elsewhere in the world. The JACMP, which has a worldwide audience of clinical medical physicists, is an appropriate vehicle for presenting a discussion of this nature.

The medical physics PD degree involves the satisfactory completion of a program consisting of didactic course work along with intensive clinical training. Upon completion of this program, the graduate has both the competency and the experience to sit for appropriate certification examinations, in particular, the examinations in various branches of radiological physics offered by the American Board of Radiology (ABR). (Although this program has often been called a Doctorate in Medical Physics, or DMP, this terminology has not yet been officially accepted by degree‐granting authorities, so in the subsequent discussion, the term Professional Doctorate will be used.)

The rationale for establishing a PD degree evolved as a response to the decision made by the ABR that, effective with the certification examination in 2014, all candidates for certification in radiological physics must complete a medical physics residency program accredited by the Commission on Accreditation of Medical Physics Educational Programs (CAMPEP), the North American organization charged with accreditation of medical physics programs. Prior to entering such a residency program, a medical physics trainee must have adequate didactic instruction, which is normally provided by approximately two years of formal instruction and typically leads to a master's degree. Consequently, after 2014, the minimum amount of postbaccalaureate training required to take the ABR examination will be approximately two years in a MS degree medical physics program followed by two years of clinical training in a residency program. Thus the first professional degree in medical physics, which qualifies an individual for entry into the profession, would be awarded after completion of four years of postbaccalaureate education. The PD program would formally combine the didactic class work and clinical training and award individuals completing such training with a doctorate degree. As a consequence, medical physics would follow the lead of many health‐related professions for which a 4‐year first professional degree is called a “doctorate” degree; for example, medicine (MD), dentistry (DDS), and veterinary medicine (DVM).

Establishment of the PD degree has been proposed to fulfill the training requirements that will be necessary to meet anticipated manpower needs. Reliable numbers do not exist, but estimates of medical physics manpower requirements for the US and Canada are in the range of approximately 150–250 new medical physicists per year. Present CAMPEP‐accredited residency programs produce only approximately 25 new medical physicists per year. Even if non‐CAMPEP‐accredited residencies were to achieve accreditation and new residencies were established, estimates indicate that residency programs could produce only approximately 75 new medical physicists per year, at best. One of the major stumbling blocks in the establishment of new residency programs is the perceived need to provide funding for medical physics residents. A PD degree program would reverse the revenue stream. PD students would pay tuition, which would help fund the training programs.

One advantage of providing clinical medical physicists with a PD degree over the present MS degree is the possible prestige that the title “doctor” would confer on a clinical medical physicist, given the fact that the physicist would have completed a training program of at least four years duration. Clinical medical physicists have sometimes found themselves at a professional disadvantage when they do not have the title of doctor. At many academic medical centers, physicists with MS degrees are not eligible for faculty positions or related benefits, even though they may do the same clinical work as a PhD. With a PD, they might be eligible for faculty positions and the benefits that go with the faculty position.

The proposed PD degree is different from the PhD degree in that the PhD degree is research oriented, whereas the PD is a professional degree. Both degrees require approximately two years of didactic medical physics education. A PhD degree includes the completion of a dissertation describing an extensive research project. This typically takes several years of work. The PD degree provides training for an individual to enter professional practice in medical physics. It is a professional doctorate, not an academic doctorate.

Students with the intention of clinical, rather than academic, careers might prefer the PD to the PhD degree. The PD is a shorter route toward a career in medical physics, taking four or five years of postbaccalaureate work, whereas the PhD route to clinical practice takes approximately five years plus an additional two years of residency. If an individual were to seek an academic career, the PhD would give that individual the appropriate mentored research training. Students in a PhD program would have the same 2‐year didactic instruction as those in a PD program, followed by several years of research experience. If such students wish to obtain clinical training, they would also have to go through a residency program.

A major difference between the PD program and the MS program plus residency is one of funding. Any sort of clinical training program requires instructional resources, which need to be financially supported. Residency programs have traditionally provided stipends to trainees. Partial cost recovery is obtained from healthcare reimbursements and additional cost recovery occurs by requiring residents to perform clinical tasks. The funding stream in a PD program is reversed; the students pay tuition to the sponsoring institution, and this tuition can be used to offset the additional costs of instruction. The PD program is designed to be a self‐sustaining method of training medical physicists, similar to the way that medical students, dental students, or veterinary students pay tuition for their professional training programs.

The financial rewards for the fully trained medical physicist are significant. Starting salaries for medical physicists in the United States are typically greater than those for dentists and veterinarians, and comparable to those for primary care physicians; dental students, veterinary students, and medical students have had no issues with paying for four years of tuition for their professional training.

A concern that some have had regarding the establishment of a PD program is the need for medical physics graduate students to become involved in some sort of research project. The argument is that a research project trains the student to become a problem‐solver, which is one of the clinical skills often expected of a medical physicist. Many present MS programs require a research project, and there is no reason why a PD program could not require the same of its students. Unless CAMPEP mandates research as part of the PD curriculum, the question of inclusion of research would be a decision made by the individual graduate programs.

Another concern that has been raised is that the PD degree might lessen the status of a PhD degree, but that really depends on the individuals holding the degrees. An individual with a PD would likely be more qualified to provide clinical support in an imaging center or radiation oncology clinic than an individual with a PhD who did not have ABR certification. An individual with a PhD is likely to be more qualified to pursue a research program.

It is important to note the potential consequences that initiating a PD program could have on other types of medical physics training programs. For example, demand among applicants for MS degree programs in medical physics may be reduced or even eliminated. If a PD program requires the same amount of work as an MS program plus a residency, there may not be incentive for a student to enter an MS program. Another issue that will need to be addressed by the medical physics community is what to do with the very large number of fully qualified clinical medical physicists already holding MS degrees. Would these individuals need to obtain PD degrees, and if so, what grandfathering mechanism could be provided for them? Although this is a new issue for the medical physics community, it is one that has been faced by other professions that have transitioned from a master's degree to a PD as the first professional degree.

Initiation of a PD program may also reduce the applicant demand for PhD programs. Many students presently enter PhD programs with primarily clinical goals in mind, and more employment opportunities exist for medical physicists in clinical, rather than academic settings. Students wishing to compete for these clinical positions would more likely apply to PD programs, rather than PhD programs. However, students who apply to PhD programs would be more likely committed to academic careers and go the route of a PhD degree followed by a residency.

Unlike the PD degree, the path of PhD followed by residency is likely to provide students with full funding during their education. The possibility exists, however, of combined PD/PhD programs, analogous to presently available combined MD/PhD programs. Students in such programs might spend 2 years taking didactic instruction, followed by 3–4 years of research, followed by 2 years of clinical training.

The presence of PD programs may also have an effect on medical physics residency programs. In the future, residency programs may be the conduit to enter the field of medical physics only for individuals with PhDs in medical physics or other disciplines. The presence of PD programs may have an even more profound effect on postdoctoral programs. Presently, participation in a postdoctoral program is a major mechanism for individuals with PhD degrees in other disciplines to enter medical physics. If a residency is required to enter the medical physics profession, applicant demand for postdoctoral positions may decrease. If only a few residency programs are available, postdoctoral fellows may not be able to compete successfully against with medical physics PhDs who have had 2 years of didactic background. If many residency programs are available, one might not need the postdoctoral experience to compete successfully for entry into a residency position. It is possible that postdoctoral positions might be filled only with individuals who want the additional research opportunities provided by a postdoctoral appointment, but that might not be such a bad idea.

In that past year, many have debated the question as to whether or not the PD is good for the medical physics profession. I believe that this is no longer the question as several institutions are currently in the process of developing PD programs. The PD will happen. What is important is that our profession institute it in a manner that will improve the quality of clinical medical physicists entering the profession. Finally, we need to remember that we are not the first profession to initiate a PD. Other professions have done so, and we have the opportunity to learn from them.

George Starkschall, PhD

Editor‐in‐Chief

May 15, 2008

